# Cyclin-Dependent Kinase 4/6 Inhibitors Plus Endocrine Therapy versus Endocrine Therapy Alone for HR-Positive, HER-2-Negative Early Breast Cancer: Meta-Analysis of Phase III Randomized Clinical Trials

**DOI:** 10.3390/jpm14050464

**Published:** 2024-04-27

**Authors:** Francisco Cezar Aquino de Moraes, Gustavo de Oliveira Almeida, Vinícius Freire Costa Alves, Jonathan N. Priantti, Giovanna da Conceição Gomes, Sarah Vitória Bristot Carnevalli, Thiago Madeira, Maysa Vilbert, Carlos Stecca, Maria Cristina Figueroa Magalhães, Marianne Rodrigues Fernandes, Ney Pereira Carneiro dos Santos

**Affiliations:** 1 Oncology Research Center, Federal University of Pará, Belem 66073-005, Brazil; 2 School of Medicine, Federal University of Triângulo Mineiro, Uberaba 38025-180, Brazil; 3 School of Medicine, University of São Paulo, São Paulo 01246-903, Brazi; 4 School of Medicine, Federal University of Amazonas, Manaus 69080-900, Brazil; jonathan.priantti@gmail.com; 5 School of Medicine, Catholic University of Minas Gerais, Belo Horizonte 32010-025, Brazil; 6 School of Medicine, Federal University of Santa Catarina, Florianópolis 88040-900, Brazil; 7 School of Medicine, Federal University of Minas Gerais, Belo Horizonte 31270-901, Brazil; 8 Department of Medical Oncology and Hematology, Princess Margaret Cancer Centre, University Health Network, Toronto, ON M5G 2C4, Canada; 9 Mackenzie Evangelical University Hospital, Curitiba 80730-150, Brazil

**Keywords:** cancer, early-stage breast cancer, endocrine therapy, cyclin-dependent kinase 4/6 inhibitors

## Abstract

Background: Cyclin-dependent kinase 4/6 (CDK4/6) inhibitors are approved for advanced breast cancer combined with endocrine therapy (ET). The efficacy of CDK4/6 inhibitors plus ET in hormone estrogen-positive, human epidermal growth factor 2-negative (HR+/HER2−) early-stage breast cancer (esBC) is still to be confirmed. Methods: We performed a systematic review and a meta-analysis to investigate the efficacy of CDK4/6i plus ET in esBC. Main outcomes included invasive disease-free survival (iDFS), distant relapse-free survival (DRFS), and overall survival (OS). We included only phase III randomized controlled trials. We used RStudio version 4.2.3, and we considered *p* < 0.05 to be statistically significant. Results: Four studies were selected, including 14,168 patients, of which 7089 were treated with CDK4/6i plus ET and 7079 received ET monotherapy. Regarding patient characteristics, 6828 (48.2%) were premenopausal. Compared with ET alone, iDFS rates (HR 0.81; 95% CI: 0.67, 0.98; *p* = 0.034) were significantly in favor of CDK4/6 inhibitors plus ET. However, there were no significant differences in DRFS (HR 0.79; 95% CI: 0.58, 1.07; *p* = 0.132) nor OS (HR 0.96; 95% CI: 0.69, 1.35; *p* = 0.829). Conclusions: Our results show that the addition of CDK4/6 inhibitors is associated with a significant benefit for HR+/HER2− esBC patients in iDFS. More studies and longer follow-up are needed to assess overall survival benefits.

## 1. Introduction

Breast cancer (BC) incidence is estimated to achieve over 3 million new cases and 1 million deaths by 2040 [1]. This is the most frequently diagnosed cancer in women in the US, excluding nonmelanoma skin cancer, and represents 14% of all new cancers [2,3]. Early-stage BC can be cured in approximately 75% of cases, highlighting the importance of early detection and early treatment [4,5]. HR+ is the most prevalent subtype of esBC [6]. For patients with HR+ BC in both early and late stages, ET has significantly improved the prognosis [7].

In the past decade, preclinical and clinical research have focused on finding new therapeutic alternatives that can prolong or restore endocrine sensitivity, postponing the need for chemotherapy and improving the survival and quality of life of patients [8]. In this scenario, a promising category of targeted therapy known as CDK4/6 inhibitors have emerged, specifically designed to target proteins involved in cell growth and division, and are increasingly being used to manage advanced BC [9]. 

CDKs form a group of proteins that play a regulatory role in the cell cycle and cell division. CDK4 and CDK6 regulate the initial gap phase of the cell cycle (G1), as when it complexes with cyclin D, this complex phosphorylates retinoblastoma proteins, releasing E2F transcription factor, allowing for cell cycle progression to the DNA synthesis (S) phase. Frequently, deregulation of this pathway causes uncontrolled cellular proliferation in breast cancer. Currently, there are three effective CDK4/6 inhibitors commercially available: palbociclib, ribociclib, and abemaciclib [9]. 

HR+/HER2- metastatic breast cancer (MBC) treatment with CDK4/6 inhibitors combined with ET has demonstrated significant improvements in oncological outcomes, including overall survival (OS), when compared with ET alone [10,11,12,13,14,15]. A prior meta-analysis by Piezzo et al. confirmed the benefit in OS with CDK4/6 inhibitors combined with ET in MBC patients [10] versus ET alone, with a hazard ratio (HR) of 0.763 (95% CI: 0.683, 0.852), regardless of aromatase inhibitor (AI)-sensitive or -resistant status. 

These CDK4/6 inhibitors have been under study in the adjuvant setting motivated by tolerable side effect profiles and successes in the metastatic setting [10]. In HR+ BC, these drugs inhibit CDK4 and CDK6 directly and indirectly, as they are downstream of estrogen receptor alpha. This inhibition leads to cell cycle arrest, blocks immunosuppressive regulatory T cells, improves antigen presentation, and induces a senescence-like phenotype [16]. However, the effectiveness of this combination in esBC is not completely understood. In this systematic review and meta-analysis, we aim to clarify the benefits of adjuvant CDK4/6i plus ET in patients with esBC in terms of invasive disease-free survival (iDFS), distant relapse-free survival (DRFS), and OS.

## 2. Materials and Methods

### 2.1. Protocol and Registration

The present meta-analysis was made according to the guidelines of the Preferred Reporting Items for Systematic Reviews and Meta-Analysis (PRISMA) declaration and the recommendations of the Cochrane Collaboration [17]. This review was registered on the Prospective International Registry of Systematic Reviews (PROSPERO) (http://www.crd.york.ac.uk/), accessed on 1 January 2024, under number CRD42023422099. 

### 2.2. Eligibility Criteria

In this review, we included phase III RCTs evaluating CDK4/6 inhibitors (palbociclib, ribociclib, or abemaciclib) combined with ET, including AI, non-steroidal aromatase inhibitors (NSAIs), selective estrogen receptor downregulators (SERDs), selective estrogen receptor modulators (SERMs), and ovarian function suppressors (OFSs), vs. ET alone in patients with HR+/HER2- esBC (Stages I-IIIC), reporting the clinical outcomes of interest.

We excluded studies with overlapping patient populations. Non-RCTs, phase II RCTs, cohort studies, case series, case reports, and reviews were also excluded. In addition, only articles in English were included, with no restrictions on the date of publication of the included articles. Thus, we sought to answer the following question: How effective is the addition of CDK4/6 inhibitors to ET vs. ET alone for the adjuvant treatment of HR+/HER2- esBC?

### 2.3. Search Strategy

We systematically searched Pubmed, Cochrane Central, Scopus, Web of Science, and the Central Register of Controlled Trials for studies published in English. We also made adaptations to the selected databases, following the syntax rules of each database and combining terms with Boolean connectors (OR, AND), as described in Supplementary Material S1.

Aiming at the inclusion of additional studies, the references of the included articles and systematic reviews of the literature were evaluated, and an alert was established for notifications in each database, in case a study corresponding to the consultation carried out was eventually published. Those found in the databases and in the references of the articles were incorporated into reference management software (EndNote^®^; version X7; Thomson Reuters, Philadelphia, PA, USA). Duplicate articles were manually and automatically excluded (by using EndNote^®^ software). 

Titles and abstracts of articles found in the databases were analyzed independently by two reviewers (G.O.A. and G.C.G.), and in case of discrepancy between the reviewers, a third reviewer was responsible for the final decision for inclusion (F.C.A.M.). 

### 2.4. Data Extraction

The following information was extracted from each study: first author, publication year, ClinicalTrials.gov Identifier, study design, regimen details in both the experimental and control arms, allocated patients for each arm, and main patient’s characteristics (median age, menopausal status, lymph node status, and histological grade). 

The following outcomes of interest were extracted: (1) iDFS, defined as time from initiation of treatment to development of invasive disease, ipsilateral breast tumor recurrence, local or regional invasive recurrence, distant recurrence, death (from BC, non-BC, or unknown cause), contralateral invasive BC, or second primary invasive cancer (non-BC); (2) DRFS, defined as the time from treatment to the date of the first distant recurrence, death (any cause), or second non-invasive primary BC; (3) OS, defined as the length of time, since the start of treatment, the patients were still alive.

Where available, the full protocol of each study was consulted to verify study objectives, population, and other relevant information regarding study design and conduct. For publications reporting results from the same study, the most recent or complete publication reporting the outcomes of interest was considered.

### 2.5. Risk-of-Bias Assessment

The Cochrane risk-of-bias tool for RCTs (RoB2) was the tool used to assess the risk of bias. It was independently analyzed by two researchers (G.O.A. and V.F.C.A.). In the case of disagreement, a third reviewer was responsible for the final opinion (F.C.A.M.). RoB2 is structured into a fixed set of domains of bias, focusing on different aspects of trial design, conduct, and reporting [18]. There are five RoB2 domains: bias arising from the randomization process, bias due to deviations from the intended interventions, bias due to missing outcome data, bias in measurement of the outcome, and bias in selection of the reported results [19].

We adopted the GRADE approach to assess the overall quality of the evidence obtained by the included RCTs [20]. GRADE categorizes evidence as being of very low, low, moderate, or high quality based on the assessment of the methodological limitations, inconsistency, imprecision, indirectness, and publication bias. For this evaluation, we used GRADEpro GDT software (Copyright © 2020, McMaster University and Evidence Prime Inc., USA).

### 2.6. Statistical Analysis

The meta-analyses were performed in R studio version 4.2.3 (R Foundation for Statistical Computing), by using the “meta” and “metafor” packages [21]. HR was used to analyze iDFS, DRFS, and OS. We considered HR for iDFS, DRS, and OS as HR > 1 favoring ET alone and HR < 1 favoring the CDK4/6 plus ET group. I^2^ was used to assess heterogeneity; a *p*-value lower than 0.10 and I^2^ > 25% were considered significant for heterogeneity. The Sidik–Jonkman estimator was used to calculate the tau^2^ variance among studies [22]. We used DerSimonian and Laird random effects models for all endpoints [23]. *p* < 0.05 was considered statistically significant. Publication bias was assessed for the main outcomes by visual analysis of the study distribution in funnel plots.

## 3. Results

### 3.1. Study Selection and Characteristics

**Figure 1:**
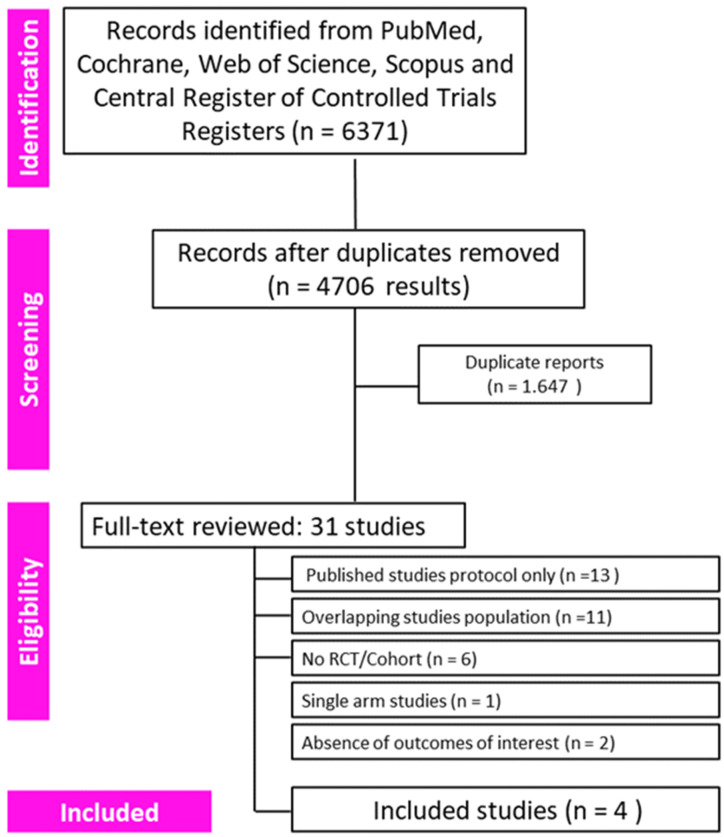
Flow diagram with systematic review and meta-analysis (PRISMA) report items. The flow diagram depicts the flow of information though the different stages of the systematic review.

The search strategy identified a total of 6371 records. After removing overlapping titles, 4706 records were evaluated by title and abstract, and of these, 31 records were eligible for full-text reading. After the analysis and application of the considered eligibility criteria, 27 articles were excluded due to not meeting the inclusion criteria, and the remaining 4 were considered for the quantitative analysis. A PRISMA flow diagram of our systematic review is shown in Figure 1. Four randomized controlled trials (RCTs) were finally enrolled in this meta-analysis [24,25,26,27,28]. 

The four studies comprised 7089 patients treated with ET combined with CDK4/6 inhibitors and 7079 patients treated with ET alone or ET combined with placebo. The minimum age of the patients was 19 years, and the maximum was 90 years. Moreover, there were 6828 (48.2%) patients with premenopausal status and 7298 (51.5%) with postmenopausal status. The most prevalent BC histological subtype was ductal, with 1103 (88.2%) patients, followed by the lobular subtype, with 110 patients (8.8%). Only one trial contributed with the histopathological features, the Penelope-B trial. In addition, the most prevalent lymph node status was one nearby lymph node containing cancer (N1), with 3682 (52.6%) patients, followed by two nearby lymph nodes with cancer cells (N2), with 1571 (15.4%). The remaining main characteristics of the enrolled studies are summarized in Table 1.

The Ki-67 levels in the Penelope-B study showed that both the control and intervention groups had 74.5% of patients with Ki-67 levels inferior to 15% [24]. Conversely, in the monarchE study, patients in the intervention and control groups had Ki-67 levels inferior to 20% only in 23.6% and 24.1% of cases, respectively [25]. Similarly, in the NATALEE study, patients with Ki-67 inferior or equal to 20% were 6.3% in the intervention group and 7.7% in the control group [26].

Among the included studies, AIs and selective estrogen receptor modulators (SERMs) were used in 67.2% and 32.5% of patients, respectively, in the PALLAS trial, and 21.6% received concurrent ovarian function suppressors (OFSs) [24]. In the Penelope-B study, 49.8% of both intervention and control groups received SERMs (with or without OFSs), while 50.2% were given AIs (with or without OFSs), and 17.1% and 18.3% of the intervention and control groups, respectively, received additional ovarian ablation [24]. In the monarchE trial, 30.9% and 69.1% of the intervention group were given SERMs and AIs, respectively, while 32.5% of the control group received SERMs and 67.6% received AIs [26]. Non-steroidal AIs (NSAIs) were used in the NATALEE trial [27].

### 3.2. Invasive Disease-Free Survival

**Figure 2:**
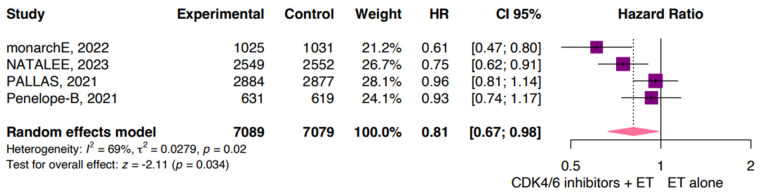
Invasive disease-free survival (iDFS). CDK, cyclin-dependent kinase; CI, confidence interval; ET, endocrine therapy; HR, hazard ratio.

According to the data available from four studies for iDFS, our pooled analysis showed a significant reduction in invasive disease for patients treated with CDK4/6i plus ET compared with those treated with ET alone (HR 0.81; 95% CI: 0.67, 0.98; *p* = 0.034; I^2^ 69%; Figure 2). The high heterogeneity is likely related to the different drugs used in each study. 

### 3.3. Distant Relapse-Free Survival

**Figure 3:**
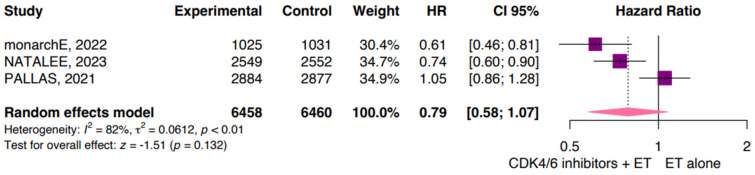
Distant relapse-free survival (DRFS). CDK, cyclin-dependent kinase; CI, confidence interval; ET, endocrine therapy; HR, hazard ratio; iDFS, invasive disease-free survival.

According to the data available from three studies for iDFS, our pooled analysis showed no significant differences in distant disease for patients treated with CDK4/6i plus ET compared with those treated with ET alone (HR 0.79; 95% CI: 0.58, 1.07; *p* = 0.132; I^2^ 82%; Figure 3). The high heterogeneity is likely related to the different drugs used in each study. 

### 3.4. Overall Survival

**Figure 4:**
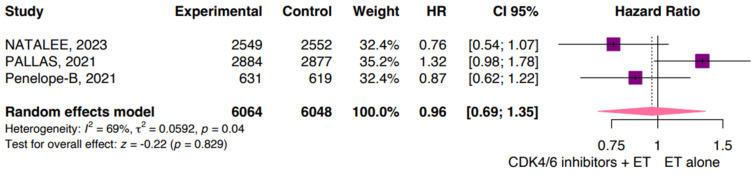
Overall survival. CDK, cyclin-dependent kinase; CI, confidence interval; ET, endocrine therapy; HR, hazard ratio.

According to the data available from three studies for OS, our pooled analysis showed a significant reduction in survival for patients treated with CDK4/6i plus ET compared with those treated with ET alone (HR 0.96; 95% CI: 0.69, 1.35; *p* = 0.829; I^2^ 69%; Figure 4). The high heterogeneity is likely related to the different drugs used in each study. 

### 3.5. Sensitivity Analyses

We performed a leave-one-out sensitivity analysis for all outcomes. First, there was a significant difference, when omitting monarchE in iDFS versus overall analysis, in heterogeneity, with the I^2^ values being 50% and 69%, respectively. Regarding DRFS (overall HR = 0.79; 95% CI: 0.58, 1.07; I^2^ = 82%), the decrease both in HR and I^2^ values happened when PALLAS was omitted (HR = 0.69; 95% CI: 0.58, 0.83, I^2^ = 14%), of which the last result achieved statistical significance. Finally, when PALLAS was removed from the OS analysis, there was a noticeable difference, with heterogeneity decreasing significantly from the overall I^2^ value of 69% to 0%. The leave-one-out sensitivity analysis of the main outcomes is detailed in Supplementary Materials S2–S4.

### 3.6. Quality Assessment

**Figure 5:**
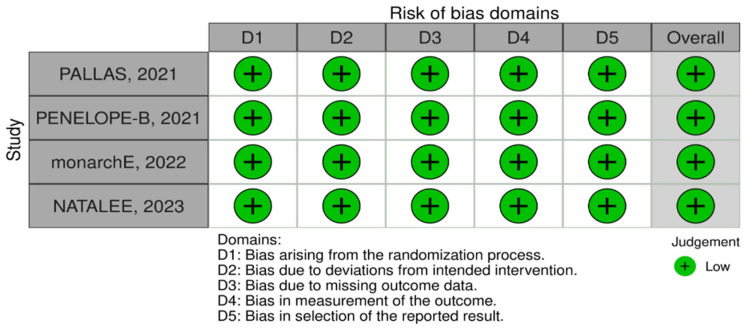
Critical appraisal of RCTs according to the Cochrane Collaboration’s tool for assessing risk of bias in RCTs.

**Figure 6:**
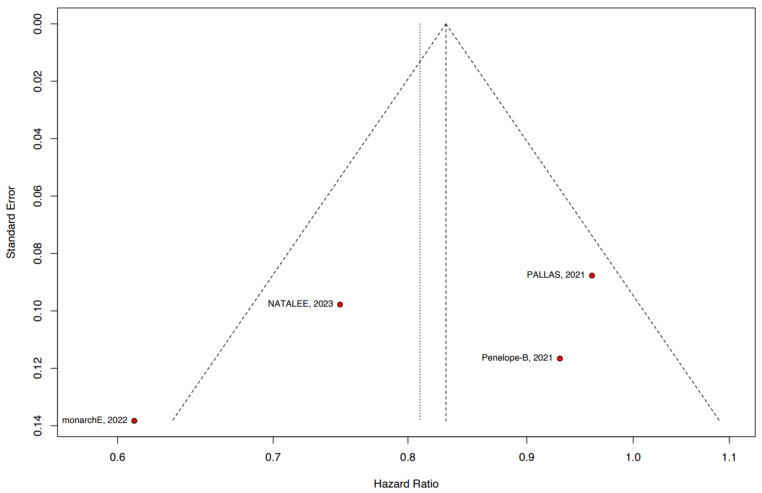
Funnel plot analysis of iDFS.

The individual assessment of each RCTs included in this meta-analysis is depicted in Figure 5. Overall, all RCTs were at low risk of bias. The funnel plot distribution in Figure 6 represents the iDFS analysis (iDFS: t-value = −1.23; degrees of freedom, df = 2 and *p* = 0.3426). In Supplementary Material S5, we present the funnel plot for DRFS (t-value of −1.07, df = 1, *p* = 0.4794), and in Supplementary Material S6, for OS (t-value of −4.54, df = 1, *p* = 0.1379).

According to the Grading of Recommendations, Assessment, Development, and Evaluations (GRADE) assessment, iDFS combined data from four RCTs, while both DRFS and OS were based on the outcomes of three trials. These results were classified as moderate-quality evidence. The GRADE quality assessment is detailed in Supplementary Material S7.

## 4. Discussion

Luminal or hormone receptor-positive (HR+) BCs without HER2 amplification or overexpression (HER2-) represent about 65–70% of all BCs [28,29,30]. The first-line therapy is currently a combination of CDK4/6 inhibitors and ET and can be divided into categories such as AIs, selective estrogen receptor downregulators (SERDs), SERMs, and OFSs [24]. For this tumor subtype, the use of chemotherapy is reserved for situations where there is a need for a high tumor response rate or if there are contraindications to the use of ET, as it presents as an indispensable measure to decrease the risk of relapse in the adjuvant setting [28,29,30,31,32,33,34]. However, despite recent advances, the recurrence rate remains at 10–25% in the first 5 years and 30–40% in later years [35,36,37,38,39,40]. 

This meta-analysis provides evidence that the addition of CDK4/6 inhibitors to ET is responsible for a significant reduction in iDFS compared with the control arm, with an HR of 81% (95% CI: 0.67, 0.98; *p* = 0.034). The monarchE trial achieved the most favorable HR for the intervention group (HR 0.61; 95% CI: 0.47, 0.80), followed closely by the NATALEE study, which also showed a favorable HR of 0.75 (95% CI: 0.62, 0.91) [31,32]. On the other hand, the PALLAS (HR 0.96; 95% CI: 0.81, 1.14) and Penelope-B (HR 0.93; 95% CI: 0.74, 1.17) trials did not yield statistically significant results in iDFS [27,28]. One possible factor contributing to these findings could be the choice of CDK4/6 inhibitors used in each study. The monarchE and NATALEE trials employed abemaciclib and ribociclib, respectively, while palbociclib was the molecule utilized in the other trials [31,32].

Abemaciclib distinguishes itself from other CDK4/6 inhibitors due to its unique pharmacokinetic and pharmacodynamic features [41,42]. Unlike palbociclib and ribociclib, abemaciclib has additional targets beyond CDK4 and CDK6, including CDK2 and CDK1 (which directly increase cell cycle progression), CDK9 (which enhances MYC activity), and GSK3a/b (which inhibits WNT signaling) [43,44]. Consequently, in the adjuvant setting, abemaciclib’s secondary targets may mediate further anti-tumor effects. Furthermore, the continuous dosing of abemaciclib may be crucial to inhibiting the activation of micrometastases, highlighting the potential significance of uninterrupted treatment regimen over intermittent dosing [41].

In addition, the monarchE trial indicated that the addition of abemaciclib to ET significantly improved iDFS at 4 years [31]. The iDFS rate with abemaciclib and ET was reported as 85.8% compared with 79.4% with ET alone, demonstrating a statistically significant benefit (HR 0.66, *p* < 0.00010) [45]. In contrast, the Penelope-B trial demonstrated that although the iDFS curves showed a slight separation at 6 months, they eventually converged after 3–4 years. Specifically, the 4-year iDFS rate was reported as 73.0% for the group receiving palbociclib plus ET compared with 72.4% for the placebo plus ET group (*p* = 0.53) [28]. 

The PALLAS trial yielded similar results. It found out that the duration of palbociclib treatment, higher dose intensity and a weighted per-protocol analysis did not lead to improved iDFS [7,23]. Both the second interim analysis at 24 months and the final analysis at 31 months showed no significant differences in iDFS between the palbociclib plus ET group and the ET-only group. The 4-year iDFS rates were reported as 84.2% and 84.5%, respectively, with no statistically significant difference being observed (*p* = 0.65) [27,40].

Moreover, the monarchE, NATALEE, and PALLAS trials contributed to this review in DRFS. The monarchE trial, which investigated the addition of abemaciclib to ET, demonstrated a lower HR for DRFS compared with the PALLAS trial [27,31,32]. Specifically, the monarchE trial reported an HR of 0.61 (95% CI: 0.46, 0.81), indicating a significant reduction in the risk of distant relapse [45]. Similarly, the NATALEE trial also showed a lower HR of 0.74 (95% CI: 0.60, 0.90) for DRFS, further supporting the benefit of CDK4/6 inhibitors in depleting distant relapse [32]. In contrast, the PALLAS study reported an HR of 1.05 (95% CI: 0.86, 1.28) for DRFS, indicating no significant improvement in DRFS with the use of palbociclib [27]. This disparity in results may largely be attributed to the selection of different CDK4/6 inhibitors in the monarchE (abemaciclib) and NATALEE (ribociclib) trials, in contrast to palbociclib in the PALLAS trial [27,32,45].

Also, the difference may be due to the studies’ population disparities. Since ET plus CDK4/6 inhibitors has shown to outperform ET alone in MBC patients [11], it is reasonable to assume that these agents would also exhibit enhanced efficacy in high-risk esBC patients. Comparing the two palbociclib trials, the slightly better Penelope-B results compared with PALLAS might have been influenced by the fact that Penelope-B examined high-risk tumors, whereas PALLAS had a higher prevalence of low-risk patients. In addition, Penelope-B only examined 1 year of palbociclib, while PALLAS administered it for 2 years, illustrating that even with a shorter drug administration period, the difference of the outcomes was not statistically significant, with only a subtle difference in favor of the intervention group in Loibl et al.’s study [7,28]. Nevertheless, it is important to note that the monarchE trial, despite having a higher proportion of low-risk-stage patients than the Penelope-B study, still showed significant improvements in iDFS and DRFS with the use of abemaciclib plus ET compared with ET alone. This suggests that this CDK4/6 inhibitor may be effective regardless of the esBC risk status [28,31].

Furthermore, the monarchE trial demonstrated that the addition of abemaciclib resulted in an improvement in DRFS at 3 years, with rates of 88.4% versus 82.5% for abemaciclib plus ET versus ET alone, respectively (HR 0.66, *p* < 0.0001) [45]. Importantly, the magnitude of abemaciclib’s benefit for both iDFS and DRFS increased over time after the completion of the 2-year abemaciclib treatment [42]. These findings indicate that in high-risk HR+/HER2- esBC, the addition of 2 years of abemaciclib improves both iDFS and DRFS in the short-term follow-up period.

When considering OS in esBC and the additional benefits of CDK4/6 inhibitors in combination with ET, the present meta-analysis reveals challenges in analyzing this outcome due to low statistical significance at different median follow-up intervals, ranging from 19.1 to 42.8 months, in the NATALEE and Penelope-B trials, respectively [28,32]. The monarchE trial reported an updated OS analysis showing a trend towards improved OS with abemaciclib, but further analysis and longer-term follow-up are necessary to determine its significance. Similarly, the PALLAS trial did not demonstrate any significant difference in OS between the groups receiving palbociclib plus ET and ET alone, with a slight difference favoring the control group [27,31]. The data from this trial indicated no significant advantage in OS for patients treated with palbociclib.

In the Penelope-B trial, OS was not statistically significant, slightly in favor of the intervention group [27]. Similarly, the NATALEE trial, despite not demonstrating a statistically significant result in OS, favored the control group, indicating that the addition of ribociclib to ET did not provide a survival advantage in this trial [28]. Further research and longer-term follow-up are needed to better understand the effects of CDK4/6 inhibitors on OS outcomes in this patient population.

It is also worth mentioning that when it comes to MBC, the benefits of using palbociclib (PAL) in first-line therapy were not as significant as shown in esBC OS. These findings are based on the following studies: PALOMA-1—median OS of 37.5 months for the combination arm versus 33.3 months for the control one; PALOMA-2—59.3 months for the combination arm of PAL + LET versus 51.2 months in the +LET- placebo group, though OS was not a primary endpoint [13]. The MONALEESA-2 and MONALEESA-7 trials reported statistically significant results for median OS and OS at 42 months, respectively, in a metastatic scenario. In the MONALEESA-2 trial, the HR for OS was 0.76 (95% CI: 0.63, 0.93), indicating a significant improvement. Similarly, in the MONALEESA-7 trial, the HR for OS at 42 months was 0.55 (95% CI: 0.44, 0.69), further supporting a significant benefit in terms of survival [14,15]. However, when using ribociclib and abemaciclib, the results regarding OS have been satisfactory [41]. 

In addition, previous studies have demonstrated the prognostic significance of Ki-67 regarding clinical outcomes in esBC, as well as its predictive value in assessing response to neoadjuvant CT or ET [45]. Patients with high-Ki-67 tumors are correlated to a greater risk of recurrence than those with low-Ki-67 tumors, which means that the Ki-67 index predicts recurrence [28,31].

For instance, subgroup analyses have demonstrated that patients derived benefit from abemaciclib regardless of their Ki-67 index or receipt of chemotherapy [31,45]. The Food and Drug Administration (FDA) approved abemaciclib in combination with ET as adjuvant treatment for HR+/HER2− esBC, specifically for patients with at least N2 disease, or N1 disease with tumor size (T) of ≥3 or grade 3 features, and a Ki-67 index of ≥20%. However, it is noteworthy that patients with a Ki-67 index below 20% still benefited from abemaciclib, as demonstrated in the monarchE trial [45]. 

Although it was difficult to compare Ki-67 status due to differences in classification (inferior or superior to 15 and 20% in the Penelope-B and both monarchE and NATALEE trials, respectively), the Penelope-B study patients had the lowest risk among the included trials, which is consistent with Ki-67 being much lower there [28,31,32].

This study has some limitations. First, the analysis was based on a restricted (limited) number of phase III randomized clinical trials with high heterogeneity, which may have influenced the effect size found in our results. However, the representativeness of the population sample in our meta-analysis conveys the best available evidence for the use of CDK 4/6 inhibitors. The heterogeneity of the results may be associated with the type of CDK 4/6 used in the studies. However, this did not prevent robust conclusions about the association’s potential for iDFS.

## 5. Conclusions

The findings of this meta-analysis provide evidence to support that adding CDK4/6 inhibitors, especially abemaciclib and ribociclib, to adjuvant ET is associated with significant benefits in iDFS for patients with esBC. At this point, no statistically significant differences in DRFS and OS were observed between the two groups; however, a longer follow-up is required for an accurate assessment of these oncological outcomes. Future studies investigating the biological behavior of each CDK4/6 are warranted to understand how possible differences in drug–tumor interaction between those agents may lead to variations in RCTs results.

## Figures and Tables

**Table 1:** Study characteristics. I, intervention experimental group; C, control group; †, median; Tx, treatment; Y, years; Mo, months; W, weeks; N/A, not available; RCT, randomized controlled trial; ET, endocrine therapy; No., number; Pre-M, premenopausal; Post-M, postmenopausal; Unk, unknown; Palbo, palbociclib; Abema, abemaciclib; PBO, placebo; Ribo, ribociclib; NSAIs, non-steroidal aromatase inhibitors. * (Minimum follow-up—Maximum follow-up), in months and ** in years. *** Months of CDK4/6 administration.

Study/Year	Design /NCT	No. of pts	Median * Follow-Up	Median Age **	Pre-M Status	Post-M Status	Tx Duration ***	Pathological Grade	Lymph Node Status	Tx	Central Ki-67
PALLAS 2021	RCT Phase III 2513394	I: 2884 C: 2877	31 mo (24.5–37.3)	I: 55 (29–84) C: 53 (30–85)	I: 1303 (45.2%) C: 1323 (46.0%)	I: 1562 (54.2%) C: 1534 (53.3%)	12	I: I or IIA: 513 (17.8) IIB or III: 2370 (82.2) Unk: 1 (0) C: I or IIA: 519 (18) IIB or III: 2358 (82) Unk: 0 (0)	I: N0: 365 (12.7) N1:1421(49.6) N2: 700 (24.3) N3: 386 (13.4) NX: 1 (0.0) Unk: 1(0) C: N0: 385 (13.4) N1:1411(49.0) N2: 709 (24.5) N3: 372 (12.9) NX: 1 (0.0) Unk:1(0)	I: Palbo + ET C: ET alone	N/A
PENELOPE-B 2021	RCT Phase III 1864746	I: 631 C: 619	42.8 mo	I: 49 (22–76) C: 48 (19–79)	I: 300 (47.5%) C: 331 (52.5%)	I: 316 (51.1%) C: 303 (48.9%)	†11.34	N/A	I: N0: 66 (10.5) N1: 433 (68.6) N2: 80 (12.7) N3: 52 (8.2) C: N0: 71 (11.5) N1: 417 (67.4) N2: 82 (13.2) N3: 49 (7.9)	I: Palbo + ET C: PBO of Palbo + ET	I: ≤15%:470(74.5) >15%:161(25.5) C: ≤15%:461(74.5) >15%:158(25.5)
MonarchE 2022	RCT Phase III 3155997	I: 1025 C: 1031	19 mo (15.6–23.9)	I: 49 (25–84) C: 49 (22–78)	I: 512 (50.1%) C: 510 (49.9%)	I: 516 (50.0%) C: 515 (50.0%)	24	I: IA: 2 (0.1) IIA: 323 (11.5) IIB: 389 (13.9) IIIA: 1027 (36.6) IIIB: 104 (3.7) IIIC: 950 (33.8) C: IA: 1 (0.0) IIA: 353 (12.5) IIB: 387 (13.7) IIIA: 1024 (36.2) IIIB: 91 (3.2) IIIC: 962 (34.0)	I: N0: 3(0.3) N1−3:450(44) N4+:572(55.8) C: N0: 6(0.6) N1−3:463(45) N4+:562 (54.5)	I: Abema + ET C: ET alone	I: <20%:242(24) ≥20%:450(44) N/A:333 (32.5) C: <20%:248(24) ≥20%:452(44) N/A:331 (32.1)
NATALEE 2023	RCT Phase III 3701334	I: 2549 C: 2552	19.1 mo	I: 52 (24–90) C: 52 (24–89)	I: 1126 (44%) C: 1132 (44%)	I: 1423 (56%) C: 1420 (56%)	36	I: IIA: 479 (19) IIB: 532 (21) III: 1528 (60) C: IIA: 521 (20) IIB: 513 (20) III: 1512 (59)	N/A	I: Ribo + NSAIs C: NSAIs alone	I: ≤20%:76 (6.3) >20%:82 (8.9) C: ≤20%:95 (7.7) >20%:15 (11.2)

## Data Availability

Data are contained within the article and Supplementary Materials.

## Supplementary Materials

The following supporting information can be downloaded at: https://www.mdpi.com/article/10.3390/jpm14050464/s1, Supplementary Material S1: search strategies; Supplementary Material S2: leave-one-out sensitive-analysis of invasive disease-free survival; Supplementary Material S3: leave-one-out sensitive-analysis of distant relapse-free survival; Supplementary Material S4: leave-one-out Sensitive-Analysis of Overall Survival; Supplement Material S5: funnel plot of distant relapse-free survival; Supplement Material S6: funnel plot of overall survival; Supplementary Material S7: GRADE analysis.
