# Supplementary material for: Cyclin-Dependent Kinase 4/6 Inhibitors Plus Endocrine Therapy versus Endocrine Therapy Alone for HR-Positive, HER-2-Negative Early Breast Cancer: Meta-Analysis of Phase III Randomized Clinical Trials
Source: J Pers Med. 2024 Apr 27;14(5):464. doi: 10.3390/jpm14050464 (PMC11121774; doi:10.3390/jpm14050464)
Supplement: Supplementary file 1 [file jpm-14-00464-s001.zip › jpm-2954520-supplementary.pdf]

Supplementary information

Supplementary Material S1: search strategies

Database	Search Strategy
PubMed	("breast neoplasm" OR "neoplasm, breast" OR "breast tumors" OR "breast tumor" OR "tumor, breast" OR "tumors, breast" OR "neoplasms, breast" OR "breast cancer" OR "cancer, breast" OR "mammary cancer" OR "cancer, mammary" OR "cancers, mammary" OR "mammary cancers" OR "malignant neoplasm of breast" OR "breast malignant neoplasm" OR "breast malignant neoplasms" OR "malignant tumor of breast" OR "breast malignant tumor" OR "breast malignant tumors" OR "cancer of breast" OR "cancer of the breast" OR "mammary carcinoma, human" OR "carcinoma, human mammary" OR "carcinomas, human mammary" OR "human mammary carcinomas" OR "mammary carcinomas, human" OR "human mammary carcinoma" OR "mammary neoplasms, human" OR "human mammary neoplasm" OR "human mammary neoplasms" OR "neoplasm, human mammary" OR "neoplasms, human mammary" OR "mammary neoplasm, human" OR "breast carcinoma" OR "breast carcinomas" OR "carcinoma, breast" OR "carcinomas, breast") AND ("early-stage" OR “early stage” OR "local advanced" OR "non-metastatic" OR "stage II" OR "stage III" OR "HR+" OR “HR +” OR "HER-" OR “HER -” OR “hormone receptor-positive" OR “hormone receptor positive" OR “hormone-positive” OR “hormone positive” OR “Human epidermal growth factor receptor 2-negative" OR “Receptors, Progesterone” OR “Receptors, Progestin” OR “Progestin Receptors” OR “Progesterone Receptor” OR “Progesterone Receptors” OR “Receptor, Progesterone” OR “Progestin Receptor” OR “Receptor, Progestin” OR “Receptors, Estrogen” OR “Estrogen Receptors” OR “Estrogen Receptor” OR “Receptor, Estrogen” OR “Receptors, Estrogen, Type II” OR “Estrogen Receptor Type II” OR “Estrogen Receptors Type II” OR “Estrogen Nuclear Receptor” OR “Nuclear Receptor, Estrogen” OR “Receptor, Estrogen Nuclear” OR “Receptors, Estrogen, Type I” OR “Estrogen Receptor Type I” OR “Estrogen Receptors Type I”) AND (“Cyclin-Dependent Kinase Inhibitor Proteins” OR “Cyclin Dependent Kinase Inhibitor Proteins” OR “CKI Proteins” OR “CDKI Proteins” OR “Cyclin-Kinase Inhibitor Proteins” OR “Cyclin Kinase Inhibitor Proteins” OR “Inhibitor Proteins, Cyclin-Kinase” OR “CIP-KIP Cyclin-Dependent Kinase Inhibitors” OR “CIP KIP Cyclin Dependent Kinase Inhibitors” OR “CIP-KIP CKI Proteins” OR “CIP KIP CKI Proteins” OR “CKI Proteins, CIP-KIP” OR “CIP-KIP CDKI Proteins” OR “CDKI Proteins, CIP-KIP” OR “CIP KIP CDKI Proteins” OR “INK4 Cyclin-Dependent Kinase Inhibitors” OR “INK4 Cyclin Dependent Kinase Inhibitors” OR “INK4 CDKI Proteins” OR “CDKI Proteins, INK4” OR “INK4 CKI Proteins” OR “CKI Proteins, INK4” OR “Inhibitors of Cyclin-Dependent Kinase 4 Proteins” OR “Inhibitors of Cyclin Dependent Kinase 4 Proteins” OR “Protein Kinase Inhibitors” OR “Inhibitors, Protein Kinase” OR “Kinase Inhibitors, Protein” OR “Protein Kinase Inhibitor” OR “Inhibitor, Protein Kinase” OR “Kinase Inhibitor, Protein” OR "CDK-4" OR "CDK 4" OR "cyclin-dependent kinase 4" OR "cyclin dependent kinase 4" OR "CDK-6" OR "CDK 6" OR "cyclin-dependent kinase 6" OR "cyclin dependent kinase 6" OR “Protein Kinase Inibitors" OR “Abemaciclib” OR “Palbociclib” OR “Ribociclib" OR “Cyclin-Dependent Kinase 4” OR “Cyclin Dependent Kinase 4” OR “Cdk4 Cyclin-Dependent Kinase” OR “Cdk4 Cyclin Dependent Kinase” OR “Cyclin-Dependent Kinase, Cdk4” OR “Cdk4 Protein” OR “Cdk4 Protein Kinase” OR “Protein Kinase, Cdk4” OR “p34PSK-J3 Kinase” OR “p34PSK J3 Kinase” OR “Cell Division Protein Kinase 4” OR “PSK-J3 Kinase” OR “PSK J3 Kinase” OR “Cyclin D-Dependent Kinase CDK4” OR “Cyclin D Dependent Kinase CDK4” OR “Cyclin-Dependent Kinase 6” OR “Cyclin Dependent Kinase 6” OR “Cell Division Protein Kinase 6” OR “Cdk6 Protein Kinase” OR “Protein Kinase, Cdk6” OR “CDK6 Protein” OR “PLSTIRE Protein”) AND ("Aromatase Inhibitors" OR "Inhibitors, Aromatase" OR "Aromatase Inhibitor" OR "Inhibitor, Aromatase" OR "Tamoxifen" OR "SERM" OR "SERM – selective estrogen receptor modulator" OR "SERMs" OR "Estrogen Receptor Modulators, Selective" OR "Selective Estrogen Receptor Modulator" OR "Estrogen Receptor Modulator, Selective" OR "Fulvestrant" OR "Endocrine Therapy" OR "Hormone Therapy")

Scopus	TITLE-ABS-KEY((("breast neoplasm") OR ("neoplasm, breast") OR ("breast tumors") OR ("breast tumor") OR ("tumor, breast") OR ("tumors, breast") OR ("neoplasms, breast") OR ("breast cancer") OR ("cancer, breast") OR ("mammary cancer") OR ("cancer, mammary") OR ("cancers, mammary") OR ("mammary cancers") OR ("malignant neoplasm of breast") OR ("breast malignant neoplasm") OR ("breast malignant neoplasms") OR ("malignant tumor of breast") OR ("breast malignant tumor") OR ("breast malignant tumors") OR ("cancer of breast") OR ("cancer of the breast") OR ("mammary carcinoma, human") OR ("carcinoma, human mammary") OR ("carcinomas, human mammary") OR ("human mammary carcinomas") OR ("mammary carcinomas, human") OR ("human mammary carcinoma") OR ("mammary neoplasms, human") OR ("human mammary neoplasm") OR ("human mammary neoplasms") OR ("neoplasm, human mammary") OR ("neoplasms, human mammary") OR ("mammary neoplasm, human") OR ("breast carcinoma") OR ("breast carcinomas") OR ("carcinoma, breast") OR ("carcinomas, breast")) AND (("early-stage") OR ("early stage") OR ("local advanced") OR ("non-metastatic") OR ("stage II") OR ("stage III") OR ("HR ") OR ("HR ") OR ("HER-") OR ("HER -") OR ("hormone receptor-positive") OR ("hormone receptor positive") OR ("hormone-positive") OR ("hormone positive") OR ("Human epidermal growth factor receptor 2-negative") OR ("Receptors, Progesterone") OR ("Receptors, Progestin") OR ("Progestin Receptors") OR ("Progesterone Receptor") OR ("Progesterone Receptors") OR ("Receptor, Progesterone") OR ("Progestin Receptor") OR ("Receptor, Progestin") OR ("Receptors, Estrogen") OR ("Estrogen Receptors") OR ("Estrogen Receptor") OR ("Receptor, Estrogen") OR ("Receptors, Estrogen, Type II") OR ("Estrogen Receptor Type II") OR ("Estrogen Receptors Type II") OR ("Estrogen Nuclear Receptor") OR ("Nuclear Receptor, Estrogen") OR ("Receptor, Estrogen Nuclear") OR ("Receptors, Estrogen, Type I") OR ("Estrogen Receptor Type I") OR ("Estrogen Receptors Type I")) AND (("Cyclin-Dependent Kinase Inhibitor Proteins") OR ("Cyclin Dependent Kinase Inhibitor Proteins") OR ("CKI Proteins") OR ("CDKI Proteins") OR ("Cyclin-Kinase Inhibitor Proteins") OR ("Cyclin Kinase Inhibitor Proteins") OR ("Inhibitor Proteins, Cyclin-Kinase") OR ("CIP-KIP Cyclin-Dependent Kinase Inhibitors") OR ("CIP KIP Cyclin Dependent Kinase Inhibitors") OR ("CIP-KIP CKI Proteins") OR ("CIP KIP CKI Proteins") OR ("CKI Proteins, CIP-KIP") OR ("CIP-KIP CDKI Proteins") OR ("CDKI Proteins, CIP-KIP") OR ("CIP KIP CDKI Proteins") OR ("INK4 Cyclin-Dependent Kinase Inhibitors") OR ("INK4 Cyclin Dependent Kinase Inhibitors") OR ("INK4 CDKI Proteins") OR ("CDKI Proteins, INK4") OR ("INK4 CKI Proteins") OR ("CKI Proteins, INK4") OR ("Inhibitors of Cyclin-Dependent Kinase 4 Proteins") OR ("Inhibitors of Cyclin Dependent Kinase 4 Proteins") OR ("Protein Kinase Inhibitors") OR ("Inhibitors, Protein Kinase") OR ("Kinase Inhibitors, Protein") OR ("Protein Kinase Inhibitor") OR ("Inhibitor, Protein Kinase") OR ("Kinase Inhibitor, Protein") OR ("CDK-4") OR ("CDK 4") OR ("cyclin-dependent kinase 4") OR ("cyclin dependent kinase 4") OR ("CDK-6") OR ("CDK 6") OR ("cyclin-dependent kinase 6") OR ("cyclin dependent kinase 6") OR ("Abemaciclib") OR ("Palbociclib") OR ("Ribociclib") OR ("Cyclin-Dependent Kinase 4") OR ("Cyclin Dependent Kinase 4") OR ("Cdk4 Cyclin-Dependent Kinase") OR ("Cdk4 Cyclin Dependent Kinase") OR ("Cyclin-Dependent Kinase, Cdk4") OR ("Cdk4 Protein") OR ("Cdk4 Protein Kinase") OR ("Protein Kinase, Cdk4") OR ("p34PSK-J3 Kinase") OR ("p34PSK J3 Kinase") OR ("Cyclin D-Dependent Kinase CDK4") OR ("Cyclin D Dependent Kinase CDK4") OR ("Cyclin-Dependent Kinase 6") OR ("Cyclin Dependent Kinase 6") OR ("Cell Division Protein Kinase 6") OR ("Cdk6 Protein Kinase") OR ("Protein Kinase, Cdk6") OR ("CDK6 Protein") OR ("PLSTIRE Protein")) AND (("Aromatase Inhibitors") OR ("Inhibitors, Aromatase") OR ("Aromatase Inhibitor") OR ("Inhibitor, Aromatase") OR ("Tamoxifen") OR ("SERM") OR ("SERM -- selective estrogen receptor modulator") OR ("SERMs") OR ("Estrogen Receptor Modulators, Selective") OR ("Selective Estrogen Receptor Modulator") OR ("Estrogen Receptor Modulator, Selective") OR ("Fulvestrant") OR ("letrozole") OR ("Endocrine Therapy") OR ("Hormone Therapy")))
Web of Science	(TS=("neoplasm, breast") OR TS=("breast tumors") OR TS=("breast tumor") OR TS=("tumor, breast") OR TS=("tumors, breast") OR TS=("neoplasms, breast") OR TS=("breast cancer") OR TS=("cancer, breast") OR TS=("mammary cancer") OR TS=("cancer, mammary") OR TS=("cancers, mammary") OR TS=("mammary cancers") OR TS=("malignant neoplasm of breast") OR TS=("breast malignant neoplasm") OR TS=("breast malignant neoplasms") OR TS=("malignant tumor of breast") OR TS=("breast malignant tumor") OR TS=("breast malignant tumors") OR TS=("cancer of breast") OR TS=("cancer of the breast") OR TS=("mammary carcinoma, human") OR TS=("carcinoma, human mammary") OR TS=("carcinomas, human mammary") OR TS=("human mammary carcinomas") OR TS=("mammary carcinomas, human") OR TS=("human mammary carcinoma") OR TS=("mammary neoplasms, human") OR TS=("human mammary neoplasm") OR TS=("human mammary neoplasms") OR TS=("neoplasm, human mammary") OR TS=("neoplasms, human mammary") OR TS=("mammary neoplasm,

human") OR TS=("breast carcinoma") OR TS=("breast carcinomas") OR TS=("carcinoma, breast") OR TS=("carcinomas, breast")) AND (TS=("early-stage") OR TS=("early stage") OR TS=("local advanced") OR TS=("non-metastatic") OR TS=("stage II") OR TS=("stage III") OR TS=("HR+") OR TS=("HR +") OR TS=("HER-") OR TS=("HER -") OR TS=("hormone receptor-positive") OR TS=("hormone receptor positive") OR TS=("hormone-positive") OR TS=("hormone positive") OR TS=("Human epidermal growth factor receptor 2-negative") OR TS=("Receptors, Progesterone") OR TS=("Receptors, Progestin") OR TS=("Progestin Receptors") OR TS=("Progesterone Receptor") OR TS=("Progesterone Receptors") OR TS=("Receptor, Progesterone") OR TS=("Progestin Receptor") OR TS=("Receptor, Progestin") OR TS=("Receptors, Estrogen") OR TS=("Estrogen Receptors") OR TS=("Estrogen Receptor") OR TS=("Receptor, Estrogen") OR TS=("Receptors, Estrogen, Type II") OR TS=("Estrogen Receptor Type II") OR TS=("Estrogen Receptors Type II") OR TS=("Estrogen Nuclear Receptor") OR TS=("Nuclear Receptor, Estrogen") OR TS=("Receptor, Estrogen Nuclear") OR TS=("Receptors, Estrogen, Type I") OR TS=("Estrogen Receptor Type I") OR TS=("Estrogen Receptors Type I")) AND (TS=("Cyclin-Dependent Kinase Inhibitor Proteins") OR TS=("Cyclin Dependent Kinase Inhibitor Proteins") OR TS=("CKI Proteins") OR TS=("CDKI Proteins") OR TS=("Cyclin-Kinase Inhibitor Proteins") OR TS=("Cyclin Kinase Inhibitor Proteins") OR TS=("Inhibitor Proteins, Cyclin-Kinase") OR TS=("CIP-KIP Cyclin-Dependent Kinase Inhibitors") OR TS=("CIP KIP Cyclin Dependent Kinase Inhibitors") OR TS=("CIP-KIP CKI Proteins") OR TS=("CIP KIP CKI Proteins") OR TS=("CKI Proteins, CIP-KIP") OR TS=("CIP-KIP CDKI Proteins") OR TS=("CDKI Proteins, CIP-KIP") OR TS=("CIP KIP CDKI Proteins") OR TS=("INK4 Cyclin-Dependent Kinase Inhibitors") OR TS=("INK4 Cyclin Dependent Kinase Inhibitors") OR TS=("INK4 CDKI Proteins") OR TS=("CDKI Proteins, INK4") OR TS=("INK4 CKI Proteins") OR TS=("CKI Proteins, INK4") OR TS=("Inhibitors of Cyclin-Dependent Kinase 4 Proteins") OR TS=("Inhibitors of Cyclin Dependent Kinase 4 Proteins") OR TS=("Protein Kinase Inhibitors") OR TS=("Inhibitors, Protein Kinase") OR TS=("Kinase Inhibitors, Protein") OR TS=("Protein Kinase Inhibitor") OR TS=("Inhibitor, Protein Kinase") OR TS=("Kinase Inhibitor, Protein") OR TS=("CDK-4") OR TS=("CDK 4") OR TS=("cyclin-dependent kinase 4") OR TS=("cyclin dependent kinase 4") OR TS=("CDK-6") OR TS=("CDK 6") OR TS=("cyclin-dependent kinase 6") OR TS=("cyclin dependent kinase 6") OR TS=("Abemaciclib") OR TS=("Palbociclib") OR TS=("Ribociclib") OR TS=("Cyclin-Dependent Kinase 4") OR TS=("Cyclin Dependent Kinase 4") OR TS=("Cdk4 Cyclin-Dependent Kinase") OR TS=("Cdk4 Cyclin Dependent Kinase") OR TS=("Cyclin-Dependent Kinase, Cdk4") OR TS=("Cdk4 Protein") OR TS=("Cdk4 Protein Kinase") OR TS=("Protein Kinase, Cdk4") OR TS=("p34PSK-J3 Kinase") OR TS=("p34PSK J3 Kinase") OR TS=("Cyclin D-Dependent Kinase CDK4") OR TS=("Cyclin D Dependent Kinase CDK4") OR TS=("Cyclin-Dependent Kinase 6") OR TS=("Cyclin Dependent Kinase 6") OR TS=("Cell Division Protein Kinase 6") OR TS=("Cdk6 Protein Kinase") OR TS=("Protein Kinase, Cdk6") OR TS=("CDK6 Protein") OR TS=("PLSTIRE Protein")) AND (TS=("Aromatase Inhibitors") OR TS=("Inhibitors, Aromatase") OR TS=("Aromatase Inhibitor") OR TS=("Inhibitor, Aromatase") OR TS=("Tamoxifen") OR TS=("SERM") OR TS=("SERM – selective estrogen receptor modulator") OR TS=("SERMs") OR TS=("Estrogen Receptor Modulators, Selective") OR TS=("Selective Estrogen Receptor Modulator") OR TS=("Estrogen Receptor Modulator, Selective") OR TS=("Fulvestrant") OR TS=(“letrozole”) OR TS=("Endocrine Therapy") OR TS=("Hormone Therapy"))

The Cochrane Library

(("breast neoplasm") OR ("neoplasm, breast") OR ("breast tumors") OR ("breast tumor") OR ("tumor, breast") OR ("tumors, breast") OR ("neoplasms, breast") OR ("breast cancer") OR ("cancer, breast") OR ("mammary cancer") OR ("cancer, mammary") OR ("cancers, mammary") OR ("mammary cancers") OR ("malignant neoplasm of breast") OR ("breast malignant neoplasm") OR ("breast malignant neoplasms") OR ("malignant tumor of breast") OR ("breast malignant tumor") OR ("breast malignant tumors") OR ("cancer of breast") OR ("cancer of the breast") OR ("mammary carcinoma, human") OR ("carcinoma, human mammary") OR ("carcinomas, human mammary") OR ("human mammary carcinomas") OR ("mammary carcinomas, human") OR ("human mammary carcinoma") OR ("mammary neoplasms, human") OR ("human mammary neoplasm") OR ("human mammary neoplasms") OR ("neoplasm, human mammary") OR ("neoplasms, human mammary") OR ("mammary neoplasm, human") OR ("breast carcinoma") OR ("breast carcinomas") OR ("carcinoma, breast") OR ("carcinomas, breast")) AND (("early-stage") OR ("early stage") OR ("local advanced") OR ("non-metastatic") OR ("stage II") OR ("stage III") OR ("HR+") OR ("HR +") OR ("HER-") OR ("HER -") OR ("hormone receptor-positive") OR ("hormone receptor positive") OR ("hormone-positive") OR ("hormone positive") OR ("Human epidermal growth factor receptor 2-negative") OR ("Receptors, Progesterone") OR ("Receptors, Progestin") OR ("Progestin Receptors") OR ("Progesterone Receptor") OR ("Progesterone Receptors") OR ("Receptor, Progesterone") OR ("Progestin Receptor") OR ("Receptor, Progestin") OR ("Receptors, Estrogen") OR ("Estrogen Receptors") OR ("Estrogen Receptor") OR ("Receptor, Estrogen") OR ("Receptors, Estrogen, Type II") OR ("Estrogen Receptor Type II") OR ("Estrogen Receptors Type II") OR ("Estrogen Nuclear Receptor") OR ("Nuclear Receptor, Estrogen") OR ("Receptor, Estrogen Nuclear") OR ("Receptors, Estrogen, Type I") OR ("Estrogen Receptor Type I") OR ("Estrogen Receptors Type I")) AND (("Cyclin-Dependent Kinase Inhibitor Proteins") OR ("Cyclin Dependent Kinase Inhibitor Proteins") OR ("CKI Proteins") OR ("CDKI Proteins") OR

	("Cyclin-Kinase Inhibitor Proteins") OR ("Cyclin Kinase Inhibitor Proteins") OR ("Inhibitor Proteins, Cyclin-Kinase") OR ("CIP-KIP Cyclin-Dependent Kinase Inhibitors") OR ("CIP KIP Cyclin Dependent Kinase Inhibitors") OR ("CIP-KIP CKI Proteins") OR ("CIP KIP CKI Proteins") OR ("CKI Proteins, CIP-KIP") OR ("CIP-KIP CDKI Proteins") OR ("CDKI Proteins, CIP-KIP") OR ("CIP KIP CDKI Proteins") OR ("INK4 Cyclin-Dependent Kinase Inhibitors") OR ("INK4 Cyclin Dependent Kinase Inhibitors") OR ("INK4 CDKI Proteins") OR ("CDKI Proteins, INK4") OR ("INK4 CKI Proteins") OR ("CKI Proteins, INK4") OR ("Inhibitors of Cyclin-Dependent Kinase 4 Proteins") OR ("Inhibitors of Cyclin Dependent Kinase 4 Proteins") OR ("Protein Kinase Inhibitors") OR ("Inhibitors, Protein Kinase") OR ("Kinase Inhibitors, Protein") OR ("Protein Kinase Inhibitor") OR ("Inhibitor, Protein Kinase") OR ("Kinase Inhibitor, Protein") OR ("CDK-4") OR ("CDK 4") OR ("cyclin-dependent kinase 4") OR ("cyclin dependent kinase 4") OR ("CDK-6") OR ("CDK 6") OR ("cyclin-dependent kinase 6") OR ("cyclin dependent kinase 6") OR ("Abemaciclib") OR ("Palbociclib") OR ("Ribociclib") OR ("Cyclin-Dependent Kinase 4") OR ("Cyclin Dependent Kinase 4") OR ("Cdk4 Cyclin-Dependent Kinase") OR ("Cdk4 Cyclin Dependent Kinase") OR ("Cyclin-Dependent Kinase, Cdk4") OR ("Cdk4 Protein") OR ("Cdk4 Protein Kinase") OR ("Protein Kinase, Cdk4") OR ("p34PSK-J3 Kinase") OR ("p34PSK J3 Kinase") OR ("Cyclin D-Dependent Kinase CDK4") OR ("Cyclin D Dependent Kinase CDK4") OR ("Cyclin-Dependent Kinase 6") OR ("Cyclin Dependent Kinase 6") OR ("Cell Division Protein Kinase 6") OR ("Cdk6 Protein Kinase") OR ("Protein Kinase, Cdk6") OR ("CDK6 Protein") OR ("PLSTIRE Protein")) AND (("Aromatase Inhibitors") OR ("Inhibitors, Aromatase") OR ("Aromatase Inhibitor") OR ("Inhibitor, Aromatase") OR ("Tamoxifen") OR ("SERM") OR ("SERM – selective estrogen receptor modulator") OR ("SERMs") OR ("Estrogen Receptor Modulators, Selective") OR ("Selective Estrogen Receptor Modulator") OR ("Estrogen Receptor Modulator, Selective") OR ("Fulvestrant") OR ("letrozole") OR ("Endocrine Therapy") OR ("Hormone Therapy"))
Clinical Trials	(Breast Neoplasms) AND (Cyclin-Dependent Kinase Inhibitor Proteins) AND (Endocrine Therapy) AND (Hormonal receptor)

Supplementary Material S2: leave-one-out sensitive-analysis of invasive disease-free survival

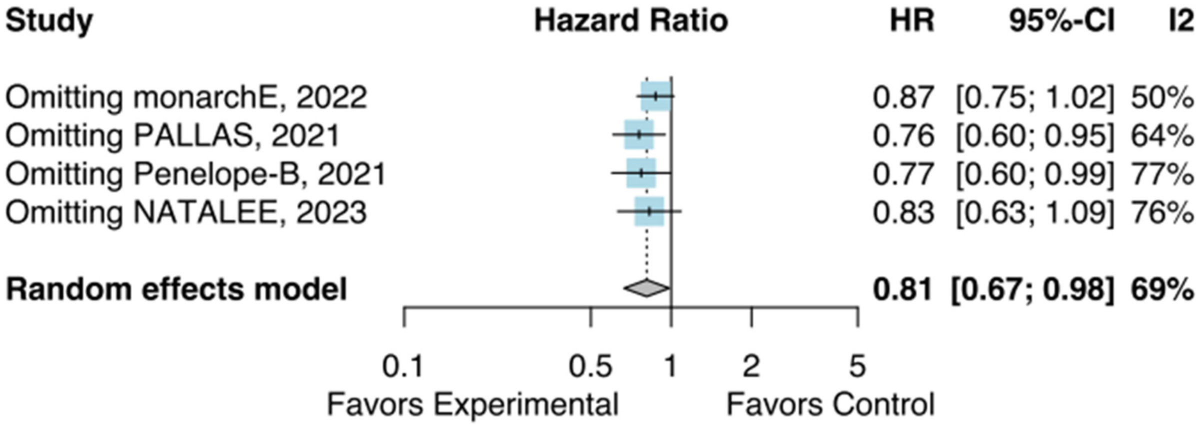

Supplementary Material S3: leave-one-out sensitive-analysis of distant relapse-free survival

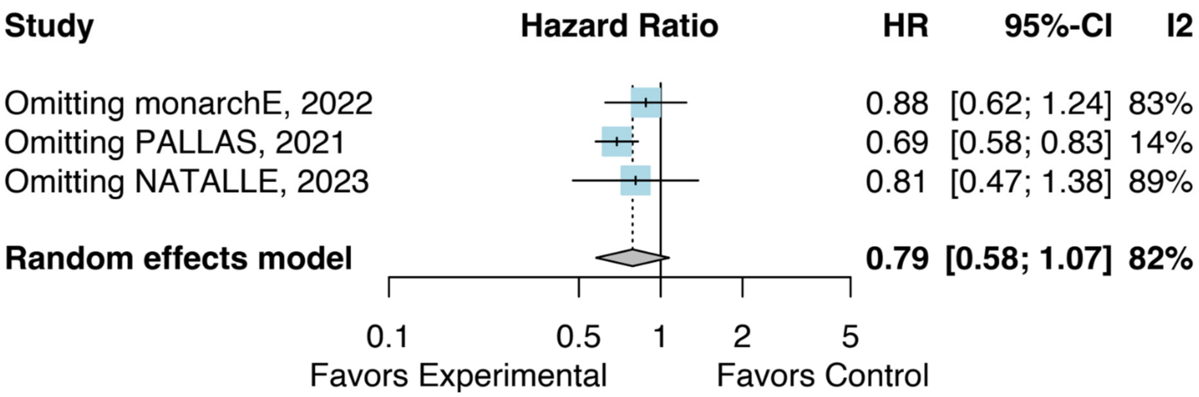

Supplementary Material S4: leave-one-out Sensitive-Analysis of Overall Survival:

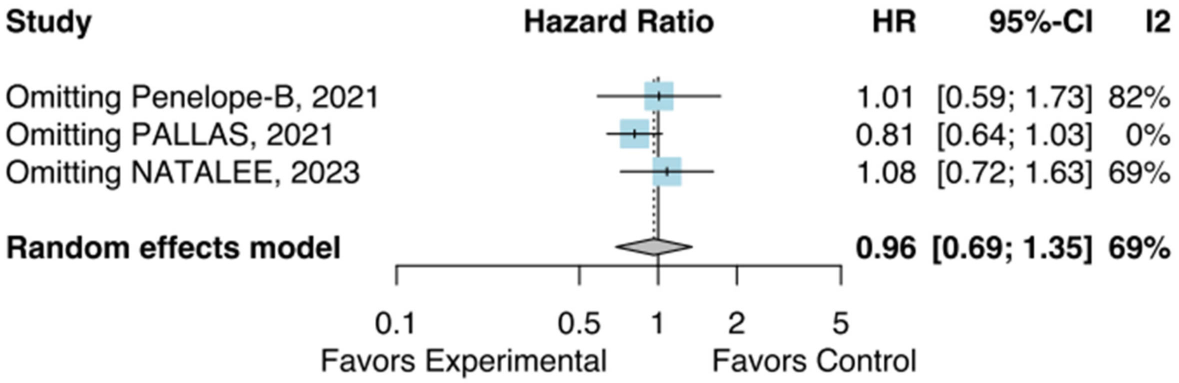

Supplement Material S5: funnel plot of distant relapse-free survival

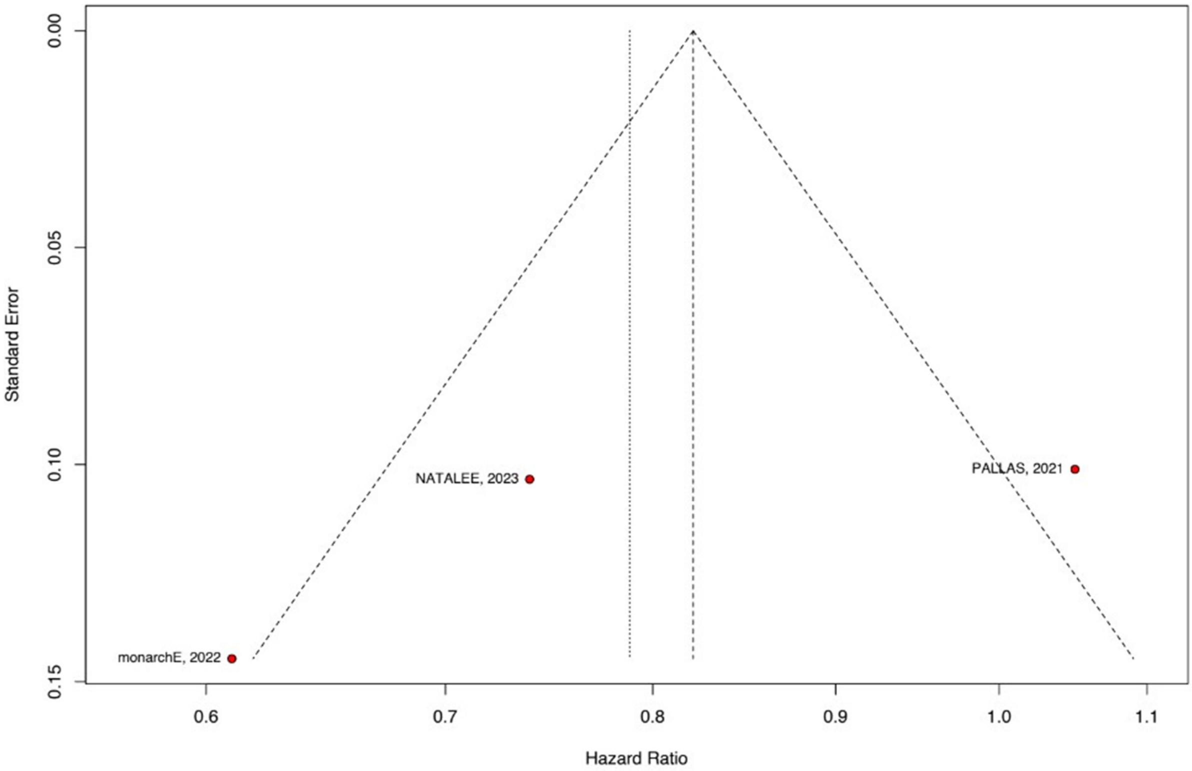

Supplement Material S6: funnel plot of overall survival

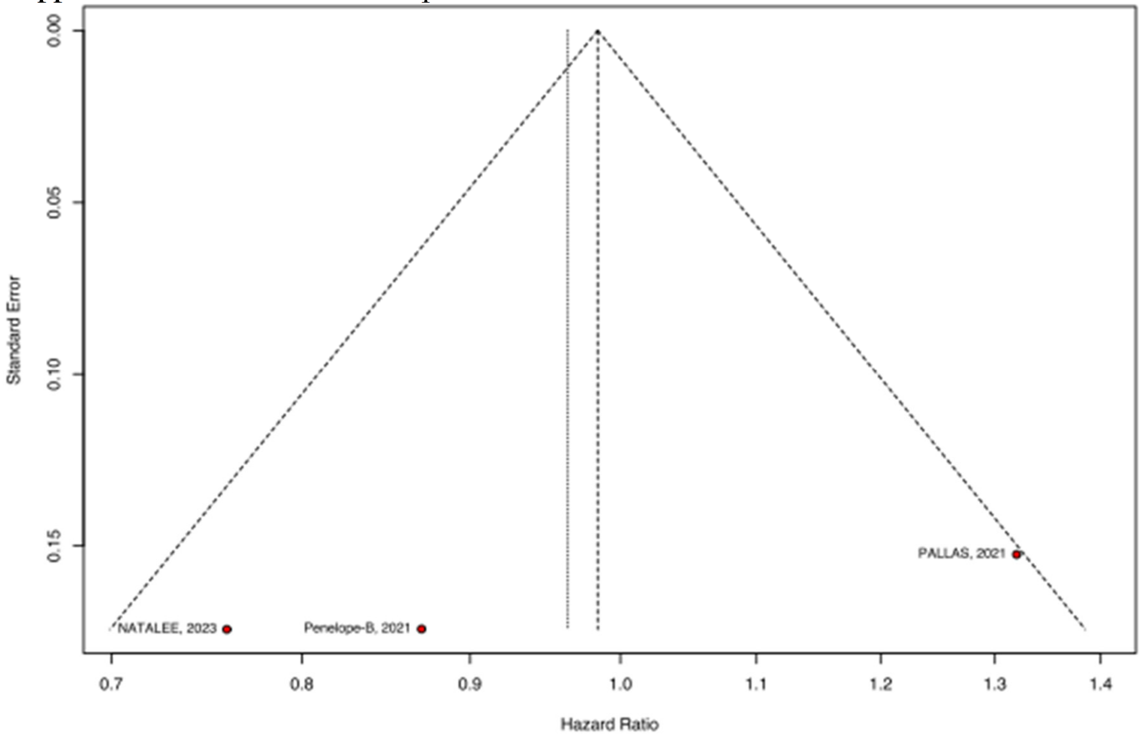

Supplementary Material S7: GRADE analysis

CDK 4/6 inhibitors + ET compared to ET alone for early ER+, HER- breast cancer

Patient or population: early ER+, HER- breast cancer
Setting: hospital
Intervention: CDK 4/6 inhibitors + ET
Comparison: ET alone

Outcomes	Number of participants (studies)	Certainty of the evidence (GRADE)	Relative effect (95% CI)	Anticipated absolute effects	
				Risk with ET alone	Risk difference with CDK 4/6 inhibitors + ET
Overall Survival (OS) assessed with: Hazard ratio	12112 (3 RCTs)	⊕⊕⊕◯ Moderate[a]	**HR 0.96** (0.69 to 1.35) [OS]	**Low**	
				80 per 100	**1 more per 100** (6 fewer to 6 more)
Invasive disease-free survival (iDFS) assessed with: Hazard ratio	14168 (4 RCTs)	⊕⊕⊕◯ Moderate[b]	**HR 0.81** (0.67 to 0.98) [iDFS]	**Low**	
				60 per 100	**6 more per 100** (1 more to 11 more)
Distant relapse-free survival (DRFS) assessed with: Hazard ratio	12918 (3 RCTs)	⊕⊕⊕◯ Moderate[c]	**HR 0.79** (0.58 to 1.07) [DRFS]	**Low**	
				52 per 100	**8 more per 100** (2 fewer to 16 more)

*__The risk in the intervention group__ (and its 95% confidence interval) is based on the assumed risk in the comparison group and the **relative effect** of the intervention (and its 95% CI).

CI: confidence interval; **HR:** hazard Ratio

GRADE Working Group grades of evidence
High certainty: we are very confident that the true effect lies close to that of the estimate of the effect.
Moderate certainty: we are moderately confident in the effect estimate: the true effect is likely to be close to the estimate of the effect, but there is a possibility that it is substantially different.
Low certainty: our confidence in the effect estimate is limited: the true effect may be substantially different from the estimate of the effect.
Very low certainty: we have very little confidence in the effect estimate: the true effect is likely to be substantially different from the estimate of effect.
